# On the role of sex differences for evolution in heterogeneous and changing fitness landscapes: insights from pygmy grasshoppers

**DOI:** 10.1098/rstb.2017.0429

**Published:** 2018-08-27

**Authors:** Anders Forsman

**Affiliations:** Ecology and Evolution in Microbial Model Systems, EEMiS, Department of Biology and Environmental Science, Linnaeus University, 391 82 Kalmar, Sweden

**Keywords:** colour polymorphism, environmental change, local adaptation, phenotypic integration, sexual dimorphism, Tetrigidae

## Abstract

Much research has been devoted to study evolution of local adaptations by natural selection, and to explore the roles of neutral processes and developmental plasticity for patterns of diversity among individuals, populations and species. Some aspects, such as evolution of adaptive variation in phenotypic traits in stable environments, and the role of plasticity in predictable changing environments, are well understood. Other aspects, such as the role of sex differences for evolution in spatially heterogeneous and temporally changing environments and dynamic fitness landscapes, remain elusive. An increased understanding of evolution requires that sex differences in development, physiology, morphology, life-history and behaviours are more broadly considered. Studies of selection should take into consideration that the relationships linking phenotypes to fitness may vary not only according to environmental conditions but also differ between males and females. Such opposing selection, sex-by-environment interaction effects of selection and sex-specific developmental plasticity can have consequences for population differentiation, local adaptations and for the dynamics of polymorphisms. Integrating sex differences in analytical frameworks and population comparisons can therefore illuminate neglected evolutionary drivers and reconcile unexpected patterns. Here, I illustrate these issues using empirical examples from over 20 years of research on colour polymorphic *Tetrix subulata* and *Tetrix undulata* pygmy grasshoppers, and summarize findings from observational field studies, manipulation experiments, common garden breeding experiments and population genetics studies.

This article is part of the theme issue ‘Linking local adaptation with the evolution of sex differences’.

## 1. Introduction

### (a) Coping with a changing world: responses to spatial and temporal heterogeneity

To understand and predict the different ways by which individuals, populations and species respond when confronted with environmental change remain key aims in ecology and evolution [1–3]. In environments that are relatively stable and homogeneous, natural selection favours those genotypes and phenotypes that are beneficial under the most prevalent conditions, resulting in evolution of specialist strategies and local adaptations [4,5]. It is also universally accepted that spatially divergent selection in combination with gene flow generally provides broad conditions for the maintenance of genetic polymorphisms [5–7].

The role of temporal environmental variation is less clear. This is partly because temporal changes may be slow and gradual, periodical and predictable, or irregular and rare. In principle, three responses are possible. Individuals may move to areas where conditions are more favourable [8]; the phenotype of individuals may show plasticity in response to environmental cues that reliably predict future selective regimes [9,10]; and the genetic architecture of populations may undergo micro-evolutionary responses to natural selection [11]. Responses and optimal solutions to temporal change may differ depending on whether the generation time of the organism is long, intermediate or short relative to the scale of the temporal changes, whether generations are discrete or overlapping and whether reproduction is semel- or iteroparous [1,6]. Variable selection in unpredictable environments may contribute to the maintenance of a diversity of specialists, promote the evolution of generalist strategies, favour diversified bet hedging strategies [12–14] or select for reversible intra-individual behavioural or physiological modifications [9,10,15].

Complexity is increased even more when environments vary in both time and space, forming dynamic mosaic landscapes. In such systems, genetic drift and rearrangements owing to abundance fluctuations and founder events driven by dispersing phenotypes may constitute important drivers of evolutionary change and contribute to population divergence, besides divergent selection and local adaptations. Dispersal may also translate into genetic admixture, the consequences of which are context-dependent and difficult to predict [16–23].

Evolution in heterogeneous environments is potentially complicated yet further by sex-specific differences. Males and females have different roles, resolve life-history trade-offs differently and are often subjected to opposing selection, leading to sex-specific genetic variation and architecture of phenotypic traits [24–31]. It remains an open question whether sex-specific differences generally constrain the evolution of local adaptations or instead preserve genetic diversity, thereby promoting the capacity to cope with challenges.

Here, I illustrate these issues building on examples from over 20 years of research on pygmy grasshoppers.

## 2. Methods

**Figure 1.**
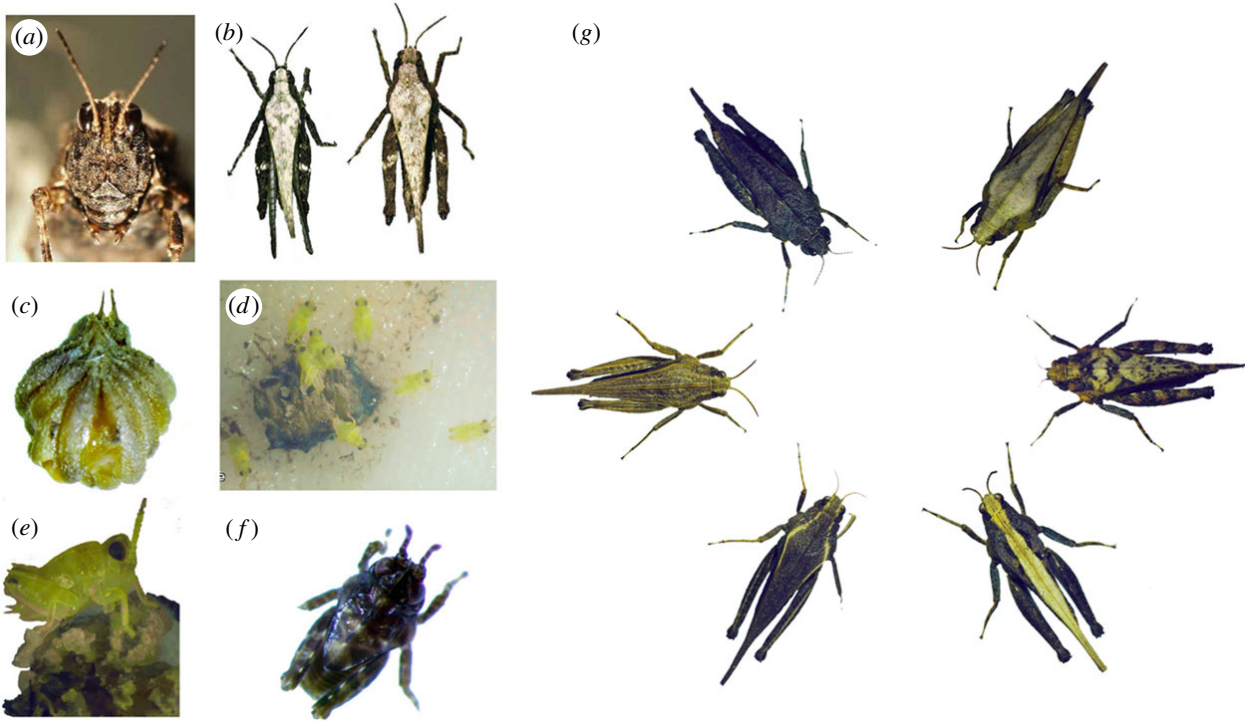
Pygmy grasshoppers. (*a*) Adult individual in frontal view, (*b*) adult male (left) and female (right) from above, (*c*) egg pod, (*d*) newly emerged hatchlings, (*e*) hatchling approximately 2 h after emerging, (*f*) hatchling approximately 24 h after emerging, (*g*) different colour morphs in adults. Photos by A.F.

Grasshoppers in the family Tetrigidae (Orthoptera: Tetrigoidea) comprise an old, cosmopolitan group of about 1500 species of small, ground dwelling, hemimetabolous insects variously referred to as ground hoppers, grouse locusts or pygmy grasshoppers, henceforth PGH [32–34]. They are characterized by the extraordinary extension of the pronotum over the entire dorsal surface of the abdomen (figure 1). They inhabit biomes ranging from tropical rainforests to arctic regions, occupy diverse habitats, are sometimes found in disturbed environments and along the shorelines of lakes, ponds and streams where they live close to the surface of the soil, feeding on algae, diatoms, detritus and mosses.

The present contribution builds on a review of previous work on *Tetrix subulata* and *Tetrix undulata* carried out by myself and collaborators, as reported in 40 original contributions in scientific journals (electronic supplementary material, table S1) and in seven compilation Doctorate theses. The findings synthesized below represent the outcomes of joint efforts and diverse approaches, including comparative field studies, capture–mark–recapture studies, phenotype and environment manipulation experiments, staged predator–prey experiments, detection experiments with humans searching for grasshopper images on computer-screens, split-brood breeding designs, stable isotope analyses, and quantitative and molecular genetics analyses. Brief outlines of the approaches used in the different investigations, together with summaries of the main findings, are included in the electronic supplementary material, table S1. More detailed accounts of the methods can be found in the original publications, included in the electronic supplementary material, table S1. At the start of this review, I conducted two topic searches, for *Tetrix subulata* and for *Tetrix undulata*, on 28 December 2017, using Web of Science. These searches generated some hits in addition to my own work that concern ecology and evolution of PGHs of relevance for issues discussed in the present review, also included in the electronic supplementary material, table S1.

## 3. Review findings

Studies of PGHs have explored behaviours (movement and dispersal, microhabitat use, foraging and diet, mating, basking, and escape and predator avoidance), physiology (thermal capacity, temperature preference, jumping performance, reaction distance, fat content and immune defence quantified as encapsulation), morphology (body size, wing size, colour pattern and developmental instability quantified as fluctuating asymmetry) and life-history traits (clutch size, inter-clutch interval, egg size, trade-off egg size versus clutch size, time to maturity, hatching success, survival of offspring, survival of adults, susceptibility to predation, prevalence of endo-parasitic fly larvae and mating success). Many of the studies have set out to determine whether phenotypic dimensions and responses differ between males and females or according to colour morph or wing morph, and whether they vary between populations and/or change over time within populations depending on environmental conditions (electronic supplementary material, table S1). Previous work also includes investigations into population performance, genetic structure and diversity (electronic supplementary material, table S1).

### (a) *Tetrix subulata* versus *T. undulata*: different species but similar patterns, processes and responses

*Tetrix undulata* and *T. subulata* represent sister taxa [35], but the genetic distance is rather high (*p*-distance: 7.8% in the mitochondrial ND1 gene [36] and 7.6% in the mitochondrial cytochrome oxidase I (COI) gene (electronic supplementary material, figure S1)), suggesting that successful hybridization is unlikely.

In my study areas in south-central Sweden, adult and late instar nymphs hibernate during winter and emerge in April–May when reproduction ensues. Females are larger than males, survive at most one reproductive season, produce multiple pods of egg (less than 35 eggs/clutch) and nymphs develop through five (males) or six (females) instars before eclosing (figure 1). Intraspecific variability is huge in both species, even among individuals within populations, in morphology, colour and pattern, physiology, behaviour and life-history (electronic supplementary material, table S1).

There are some species-specific idiosyncrasies, for instance, in how wing morph frequencies, body size, and genetic structure and diversity respond to similar environments [17]. Moreover, *T. subulata* is widely distributed in Europe, Asia and much of North America south to Mexico [32,37] and usually occupies damper microhabitats in relatively open areas (e.g. clear cuttings, shore meadows, pastures). By comparison, *T. undulata* has a more restricted distribution in Europe, usually occupies drier microhabitats, and it is more often short-winged albeit with some variation among populations [17,32,38]. These differences aside, an overall conclusion that emerges when analysing and comparing results from the large number of ecological and evolutionary investigations is that the general patterns and responses seem similar in the two species (see electronic supplementary material, table S1). This opens for replication at the interspecific level and for paired comparisons, approaches aimed at generalizations and identifying key drivers of variation and change.

### (b) Colour polymorphism and its relation to sex

One of the most distinctive features of PGHs is that they display an enormous variability in colours and patterns. Intra-specific variation in these animals has been documented and analysed for almost a century [37,39–41]. Ground colours range from black, via various shades of brown and olive green, to light grey. Some morphs are monochrome, while others have patterning consisting of longitudinal stripes, vertical bars, or specks or spots of variable colours and widths (figure 1). Morphs also vary with regard to texture of the integument on the pronotum and the femur of the jumping legs, the surface being either smooth, or granular and rough, or consisting of longitudinal ridges and grooves (veining). Variation is extensive even within morphs. The colour polymorphism is expressed in both males and females, but selection and spatio-temporal shifts in morph frequencies are sex-dependent (outlined in §4).

#### (i) Similarity among and flexibility within species

**Figure 2.**
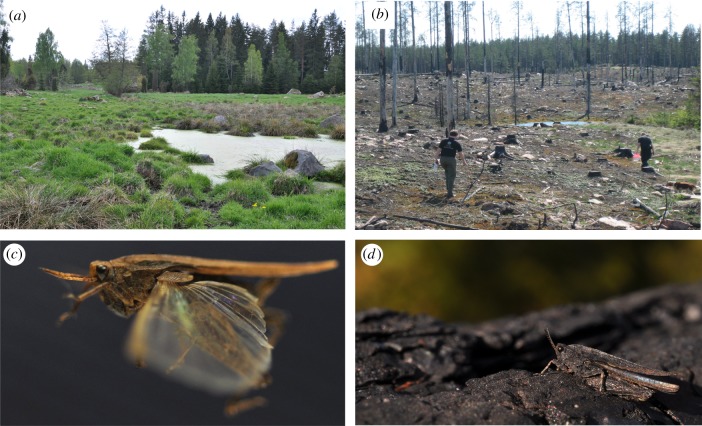
PGH often occupy environments influenced by small- or large-scale disturbances, such as (*a*) shore habitats and pastures influenced by trampling cattle, and (*b*) forest and clear-cut fields influenced by natural or managed fires. Macropterous (*c*) phenotypes with long functional wings are able to colonize, and occur in high frequency in, recently disturbed habitats. (*d*) Individuals belonging to the black morph are well camouflaged in post-fire environments and vary in frequency in time and space. Photos by A.F.

The exuberant diversity in colours and patterns is shared by most, if not all, Tetrigidae grasshoppers, pointing to the conclusion that the polymorphism is beneficial and of phylogenetic antiquity [5]. There is also considerable flexibility of the polymorphism within species. The relative frequencies of colour morphs vary among populations and change over time within populations according to spatio-temporal variation in environmental conditions [38,41–43] (Y. Yildirim, J. Tinnert, A. Forsman 2018, unpublished manuscript). This includes ‘fire melanism’ manifest as rapid evolutionary shifts in the incidence of the black form driven by oscillating selection in post-fire environments [41,43,44] (figure 2). Populations in stable environments are also less colour morph diverse than in disturbed environments [38] (Y. Yildirim, J. Tinnert, A. Forsman 2018, unpublished manuscript), possibly owing to purifying or stabilizing selection.

#### (ii) Colour patterns are inherited and not influenced by plasticity

It is common among Acridoidae grasshoppers that colour patterns are plastic and respond to conditions experienced during embryonic development and growth [45,46]. The superfamily Tetrigoidea is different in this regard, in that it contains a number of species in which the polymorphism appears to be under strong genetic control and little influenced by plasticity [39,40,47] (electronic supplementary material, table S1). For instance, the distribution of alternative colour morphs among offspring within a clutch depends on the colour pattern of the mother [43,48–50]. Families of half-sibling offspring sired by many fathers are more colour morph diverse compared with families sired by a single or a few males [50,51]. Split-brood experiments have shown that neither the patterning nor the overall darkness of pattern elements is influenced by substrate, temperature or crowding [48,49,52,53]. Colour morph frequencies in samples of wild-caught individuals from different populations are highly correlated with those in captive reared individuals, indicative of population-level heritability [41]. Hochkirch *et al.* [54] state that colour patterns in *T. subulata* are plastic. However, because the experimental protocol used was not designed to eliminate other sources of variation, there are alternative explanations to what the authors [54] interpret as plasticity, and there exists as yet no firm evidence that the colour polymorphism in PGHs is affected by developmental plasticity (for a detailed discussion, see [48]). A possible explanation as to why the colour morphs are not influenced by plasticity is that PGHs often use unstable habitats characterized by unpredictable environmental change.

#### (iii) Functional importance and selective drivers

There are many ways by which colour pattern can contribute to variation in performance and fitness among individuals, and thereby impact the spatio-temporal dynamics of colour polymorphisms [55]. There is no evidence that colour morph is used as a cue during mate choice, either by male or female PGHs [56]. Between-species interactions probably are more important. Colour patterns can offer protection against enemies by influencing the probabilities that prey individuals are detected, recognized, attacked and captured [57,58]. Their small size and locally high population densities render grasshoppers susceptible to visual vertebrate predators such as birds [59–61] and lizards [62]. Studies using different approaches indicate that colour morph shifts are driven at least in part by differential predation and selection for camouflage that varies according to sex [42,44,61–64], as outlined in §4, also in other *Tetrix* species [65,66].

Invertebrates such as spiders [67] and endoparasitic flies [62,68] may also contribute to grasshopper mortality, but probably do not select their prey on the basis of colour pattern. It has been suggested that melanistic individuals benefit from an improved physical barrier against infection, wound healing, cellular innate immunity and parasite resistance [69,70], but apparently not in PGHs [68]. Studies into how differential predation may impact the dynamics of colour polymorphism have focused on adults, and very little is known about predation on immature nymphs. This is unsatisfactory both because an important evolutionary driver may have gone undetected and because the number of instars differs between sexes.

Colour patterns of PGHs affect temperature regulation. During sun basking, darker morphs warm up faster and attain higher equilibrium body temperatures compared with paler morphs [71,72]. Such differences in thermal capacity can potentially influence lifetime reproductive success, because body temperature affects all aspects of organismal performance, ranging from physiology and locomotion to behaviour and life-history [49,61,73–75]. The evolutionary consequences of this are, however, complicated by the fact that preferred temperatures differ between males and females [76,77], as outlined in §4.

**Figure 3.**
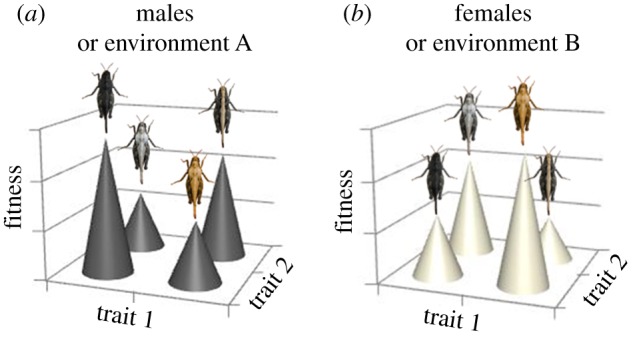
Hypothetical fitness landscapes in colour polymorphic PGH. Certain combinations of trait 1 and trait 2 (for example, body size and body temperature) can be either favourable or detrimental depending on colour pattern (or some other phenotypic dimension), resulting in evolution of alternative complex phenotypes (eco-morphs). Relative fitness (height of the peaks) of alternative trait value combinations may be different in males and females within the same environment, differ between environments and change over time within environments.

Selection imposed by predators and thermal conditions likely play key roles as drivers of clines, mosaics and dynamics of colour polymorphism in PGHs [41,63,64,72,76]. However, evolution of spatial differences and temporal shifts in morph frequencies can also be influenced by indirect responses to selection on traits that are developmentally, genetically and phenotypically associated with colour pattern [55,78,79], and by sex-specific differences in behaviour, physiology, body size and morphology (electronic supplementary material, table S1; figure 3).

### (c) Phenotypic integration

Colour morphs of PGHs represent integrated phenotypes [49,63,72,76,77,80] (electronic supplementary material, table S1 and figure 3). For example, heating rates, preferred body temperatures, thermal performance curves of bodily functions and microhabitat utilization are correlated, differ among colour morphs and vary according to maternal colour morph [72,76,77,80]. The inferior thermal capacity of paler morphs has been accompanied by coevolutionary modifications in thermal physiology and behaviours. Conversely, the superior heating rate of darker morphs may come at a cost. Owing to high absorbance of solar radiation, surface temperatures in areas covered with burnt-off material can be very high [76] and these ground-dwelling insects shuttle between microhabitats to avoid overheating [72,76,81]. Interestingly, the degree of habitat selectivity is highest in the black morph [76]. Despite this, black *T. undulata* individuals have higher fluctuating asymmetry compared to paler morphs [82], possibly because their development is perturbed by temperature stress.

PGH colour morphs vary in body size [17,49,63,83] and life-history traits such as inter-clutch intervals, the trade-off between number and size of eggs and time to maturity [49,74,83]. Morphs differ also in behaviours related to microhabitat utilization [63,72,76,84], diet [85,86] and predator avoidance [76,80].

The genetic underpinnings of this phenotypic integration remain unclear, but viable possibilities include supergenes, genetic correlations via pleiotropic effects and regulatory switch mechanisms. That morph frequencies vary between males and females (electronic supplementary material, table S1) supports this conclusion. Different colour morphs do not seem to be fully genetically compatible. Dark individuals born to dark mothers survive better than dark individuals born to paler mothers, and pale individuals born to dark mother survive worse than pale individuals born to pale mothers—indicative of co-evolved morph-specific genetic combinations [52]. Caesar & Forsman [87] also found that parental colour morph resemblance increased viability of offspring, pointing to compatibility effects. Despite this, there is no evidence for mate choice or assortative mating with regard to colour morph. Instead, PGHs are promiscuous [56,87], and females mated to several males produce offspring that are half-siblings and therefore phenotypically and genetically more diverse [39,50,51].

As we have seen, the colour polymorphism in PGHs and other animals can inform about phenotypic integration and local adaptation. In addition, it can have important population-level consequences [3,55,88–90], for example, by lessening the negative effects of intra-specific competition [91], reducing predation [92,93] and improving establishment success in novel areas [94]. But the polymorphic condition in PGHs goes beyond colour patterns, it applies also to dispersal.

### (d) Dispersal polymorphism and associations of wing morph with sex, body size, colour morph and genetic diversity

PGHs are sedentary animals that normally move only a few metres per day [56,63,95]. However, like many other insects [96,97], *T. subulata* and *T. undulata* are wing dimorphic. The wings generally correspond in length with that of the pronotum, and macropterous individuals with a long pronotum have fully developed functional wings [17,39,95,98] (figure 1), which may be beneficial in the search for mates or food [86], aid predator avoidance [61], and promote dispersal, colonization and gene flow [17,18,34,37,38,95].

The macropterous morph is more common in *T. subulata* than in *T. undulata*, as shown, for example, by pairwise comparisons in sympatry [17]. Spatial and temporal variation in the incidence of the long-winged morph is highly correlated in males and females (result based on data for *T. subulata* from [95], *r* = 0.84, *n* = 20, *p* < 0.0001) and the long-winged morph is equally common in both sexes (paired *t*-test, *n* = 20, *t* = −0.27, *p* = 0.79). The long-winged morph is equally common in males and females also in *T. subulata* populations in Germany (results based on analysis of data in table 1 in Steenman *et al.* [98], paired *t*-test, *n* = 13 samples, *t* = −1.27, *p* = 0.23).

With regard to phenotypic integration, Berggren *et al.* [95] show that there is no difference in body size (measured as length of posterior femur) between long- and short-winged individuals. Re-analysis of data collected more recently from another population [16] confirms that wing morphs do not differ in body size in our study areas (ANOVA, effect of sex: *F*_1,194_ = 852.70, *p* < 0.0001; effect of wing morph: *F*_1,194_ = 0.27, *p* = 0.60), but see Steenman *et al.* [98]. There is also no correlation between maternal body size and the incidence of long-winged phenotypes among captive-reared offspring [95], arguing against a genetic correlation between wing morph and body size. Available evidence based on analyses of individuals collected in the field indicate that wing morph is also independent of colour morph [41]. Re-analysis of data from Berggren *et al*. [91] shows that the proportion of long-winged *T. subulata* phenotypes in families reared in captivity under controlled conditions differs depending on maternal wing morph but is independent of maternal colour morph (generalized linear mixed model, maternal wing morph: *F*_1,212_ = 205.76, *p* < 0.0001; effect of maternal colour morph: *F*_5,212_ = 0.70, *p* = 0.62, electronic supplementary material, figure S2), arguing against a genetic correlation between colour morph and wing morph.

Females with functional wings have similar clutch sizes and inter-clutch intervals on average as short-winged females [95], indicating that dispersal capacity does not come at the cost of reduced reproductive output, at least not in these populations (but see [99]).

Associations of long-winged phenotypes with estimates of population genetic divergence, and with within-population genetic diversity, confirm that gene flow influences the evolution of PGH populations [17,18,38] (Y. Yildirim, J. Tinnert, A. Forsman 2018, unpublished manuscript). The combination of colour polymorphism with dispersal polymorphism means that PGHs can cope with deteriorating conditions either by rapid evolutionary modifications or by dispersing to more favourable areas.

## 4. Consequences of sex-specific differences for evolution in heterogeneous and dynamic fitness landscapes

Some species of PGHs share characteristics akin to ‘ruderal species’ of plants, in that they thrive in habitats disturbed by fires, cultivation, trampling by cattle or wave action, and seem to use a tracking strategy suitable for temporally unstable environments. Accordingly, the frequency of the long-winged morph is higher in recently disturbed than in stable environments in both *T. subulata* [38,95] and *T. undulata* [38] (Y. Yildirim, J. Tinnert, A. Forsman 2018, unpublished manuscript), indicative of immigration and establishment events. Their ecological characteristics and transient (meta-population) population dynamics make PGHs an interesting model system for studies of local adaptation and population genetic structure.

New populations can be established by small groups of only six individuals, and founder groups that are more colour morph diverse are more successful [94,100]. Their promiscuous mating behaviour [56,87] may increase effective population size of small founder groups and mitigate negative effects of inbreeding. A few founder females that have mated with multiple males may give rise to new populations that harbour much of the genetic variation in the source population [51,101]. Additionally, conversion of non-additive (epistatic and dominance) to additive genetic variance and the physical inhibition of recombination associated with chromosomal rearrangements in small populations may result in increased (not decreased) genetic variance in quantitative traits [102–107]. This can create novel phenotypes and increase evolvability [43]. Immigration may also translate into inter-population hybridization (admixture), with consequences for genetic structure and population fitness that are context-dependent and therefore difficult to predict [16–23]. Evolutionary responses to environmental changes are complicated further still by sex-specific differences in ecology, physiology, behaviour, selection and genetic background.

In the case of PGHs, sexual size dimorphism is distinct; females are larger than males (electronic supplementary material, table S1) and pass through one additional nymphal instar [32]. Females prefer higher body temperatures and seem to be more ‘picky’ with regard to temperature preferences compared with males [76,77]. The utilization of different microhabitats and substrate types differs between sexes [63,76], and females are more active and move longer distances than males [63]. Because males and females have different ecological roles, they are exposed to different selection pressures (figure 3). That this can impact on evolution is evidenced both by molecular and phenotypic data.

Ongoing work (Y. Yildirim, J. Tinnert, A. Forsman 2018, unpublished manuscript) based on analyses of DNA (amplified fragment length polymorphism, AFLP) data for *T. undulata* from 20 sampling locations indicate that genetic structure based on outlier loci influenced by direct or indirect selection differs according to population and between sexes (electronic supplementary material, table S2). On a more general level, sex-specific local adaptations can manifest as geographical variation in the degree of sexual size dimorphism [108,109], a pattern that is also evident among populations in PGHs [16].

The colour polymorphism and ‘fire melanism’ in PGHs provide another striking example of phenotypic integration, rapid evolutionary change and local adaptation in a dynamic, spatially heterogeneous and temporally changing fitness landscape that is complicated further by sex-specific differences. This can be visualized by hypothetical fitness landscapes (figure 3). In PGHs, certain trait value combinations (for example, body size and body temperature) can be either favourable or detrimental depending on colour pattern. Correlational selection has resulted in phenotypic integration and evolution of alternative complex phenotypes (eco-morphs). Relative fitness (height of the peaks in figure 3) of alternative trait value combinations may be different in males and females within the same environment, differ between environments (environment A or B in figure 3), and change over time within environments. Because of sex-related differences in morphology, physiology, behaviour and reproductive roles, spatio-temporal dynamics in the fitness landscape are not necessarily correlated in males and females. This impacts evolution of local adaptations and patterns of variation among and within populations.

Spatial variation and temporal shifts in the incidence of the black colour morph are correlated in males and females (results based on re-analyses of data for *T. subulata* [41], *r* = 0.78, *n* = 31 samples, *p* < 0.0001). This suggests that at an overall level, shared selective environments induce parallel micro-evolutionary responses in the two sexes. However, the black morph is 95% more common overall in males (paired *t*-test, *t* = 3.47, *n* = 31, *p* = 0.0016), perhaps in part because the protection against predation offered by different colour patterns depend on body size. Thus, detection experiments show that the relative crypsis of different PGH colour morphs varies depending on the visual background [44,64], and that the relative protective value of black coloration is greater for small than for intermediate and large individuals [42]. Given that male PGHs are smaller than females and that size can modify detectability (and hence presumably relative fitness) of alternative colour morphs [42], evolution might have resulted in sex-linked expression of colour pattern, as in other species [66,110]. An additional explanation as to why the black morph is more common in males might be that black males seem to have a higher mating success compared with other morphs [56]. Such a mating advantage [56] might contribute to the long-term persistence, albeit at low frequencies, of the black morph in populations in non-burnt environments where dark coloration offers no superior protection against predation [44,64].

When colour morph frequencies in PGHs are analysed at finer resolution, it becomes evident that differences among populations and temporal changes within populations are sex-specific [42]. Assuming that colour morph frequencies are driven at least partly by selection, this implies that phenotypes corresponding to local adaptations differ depending on sex. The moulding effect of sex-specific differences also manifests in body size. Body size of PGHs varies depending on both colour morph and sex, but size differences among morphs are discordant in males and females [49], indicating that the optimal solution to phenotypic integration differs between sexes within populations (figure 3).

The black and hot surfaces that characterize fire-ravaged areas before vegetation has recovered may differently influence body temperatures, water balance and camouflage depending on colour pattern, body size and sex of individuals [44,45,58,63,76,111,112]. For example, an association of thermal preference with colour pattern is evident in female but not in male PGHs [77], and the degree of small-scale microhabitat selectivity varies among colour morphs [76]. As discussed above, colour pattern is known to also influence probability of detection and predation. Work on PGHs shows that the protective value of alternative colour patterns differ between males and females according to behaviours and movement patterns [61,63], body size [42] and visual characteristics of the habitat [44,64]. That predation may impose opposing selection on colour pattern in males and females has also been reported in *Tetrix japonica* [65,66], and in lizards [110] and snakes [28,113].

In ectothermic organisms in general, spatio-temporal differences in ambient temperature and the potential for thermoregulation associated with latitude, altitude, seasonality, variable weather conditions and climate change have potential to influence behaviour, performance and fitness of individuals as well as evolution of populations and range distributions of species—but responses often depend on sex and/or colour pattern [75,84,110,114–120].

Differential selection in males and females can potentially thwart evolution of local adaptations [30]. Depending on genetic architecture, the evolutionary response to environmental change may be a compromised outcome of selection in males and females (figure 3). On the other hand, opposing selection can maintain genetic and phenotypic diversity within populations [28,121,122]. Experimental and comparative evidence agree that genetic and phenotypic diversity increases evolutionary potential and promotes ecological success of populations and species [88–90]. By preventing the erosion of genetic diversity, opposing selection in the two sexes may thus allow for faster evolutionary responses and persistence in the face of natural and human-induced environmental changes. Similarly, their promiscuous mating behaviour resulting in more diversified offspring [39,50,51] may permit, and be a consequence of, using unpredictable dynamic fitness landscapes.

## 5. Future directions

Integrating sex differences in analytical frameworks and population comparisons can help reconcile unexpected patterns and illuminate neglected evolutionary drivers. In the case of PGHs, there is opportunity for future investigations at different hierarchical levels to generate novel insights. For instance, previous studies concur that the colour and dispersal polymorphisms are genetically encoded and heritable, but the details remain to be discovered and may deepen our understanding of their evolutionary dynamics.

Analyses of outlier loci (electronic supplementary material, table S2) can potentially generate insights into sex-by-environment interactions for selection, and can also be applied to colour morphs to identify genes under selection. Recent developments in genomic tools (e.g. RAD-sequencing [123]) together with information for closely related species [124] may help elucidate the underpinnings of phenotypic integration, and clarify the contributions of stochastic processes, gene flow, selection and plasticity in shaping genetic structure and phenotypic evolution in PGHs.

Comparisons of colour morph distributions in males and females from populations in stable, disturbed and changing environments [38] (Y. Yildirim, J. Tinnert, A. Forsman 2018, unpublished manuscript) where the two species are sympatric [17] have the potential to inform about: whether spatial differences and temporal shifts in colour morph frequencies are parallel or independent in males and females, as might be expected if opposing selection and sex-specific responses prevent evolution of local adaptation; and whether evolutionary responses to environmental challenges are species-specific or shared. New insights might also be obtained by studying evolutionary transitions of polymorphisms and of sexual dimorphisms within a phylogeny-based comparative framework.

## Supplementary Material

Table S1

## Supplementary Material

Table S2

## Supplementary Material

Figure S1

## Supplementary Material

Figure S2

## Data accessibility

The data supporting this review are included in the original articles listed in electronic supplementary material, table S1.
