# Supplementary material for: On the role of sex differences for evolution in heterogeneous and changing fitness landscapes: insights from pygmy grasshoppers
Source: Philos Trans R Soc Lond B Biol Sci. 2018 Aug 27;373(1757):20170429. doi: 10.1098/rstb.2017.0429 (PMC6125723; doi:10.1098/rstb.2017.0429)
Supplement: Table S1 [file rstb20170429supp1.pdf]

Table S1. Summary of findings in studies that have investigated how behaviour, physiology, morphology, life-history, performance, and genetic structure and diversity differ according to sex, colour morph, and population in *Tetrix subulata* and *T. undulata* pygmy grasshoppers. Some studies that investigated more than one response variable have more than one entry.

Species/Trait	Differences between: Males and females	Colour morphs	Populations	Description of main finding(s)	Methodological approach used	Study
TETRIX SUBULATA						
Behaviours						
Daily dispersal distance	+	NE	NA	Females dispersed longer distances than males	Mark-recapture study	Forsman and Appelqvist [1]
Microhabitat use	+	NE	NA	Utilization of different surface substrate types varied according to sex	Mark-recapture study and behavioural observations of free-ranging individuals	Forsman and Appelqvist [1]
Microhabitat choice of manipulated phenotypes	NE	+	NA	Individuals painted black resided in microhabitats with less solar radiation compared with females painted white, females painted white produced more offspring under increased radiation (mainly due to increased mortality of black painted females)	Microhabitat choice in a thermal mosaic gradient was compared between individuals that had been painted either black or white, reproductive performance of black and white painted individuals was compared in normal and elevated solar radiation treatments	Karpestam *et al.* [2]
Diet	NA	+	+	Utilization of food types in choice experiment differed among populations and among colour morphs within populations, stable isotope ratios indicated long-term dietary differences between populations and among colour morphs within populations	Wild-caught females from five populations were compared for dietary preferences in laboratory choice trials, and realized dietary niches in the wild were quantified based on stable isotope analyses of individuals from two populations	Karpestam and Forsman [3]
Diet	-	NE	+	Nitrogen (but not carbon) stable isotope signatures (indicative of long-term dietary differences) were independent of sex but differed between wing morphs depending on source	Wild-caught macropterous (long-winged) and brachypterous (short-winged) females from two populations	Karpestam and Forsman [4]

				population	were compared for long-term realized dietary niches in the wild based on stable isotope analyses	
Mating behaviour and mating success	NA	+	NA	Both males and females mated with multiple mates, no evidence for assortative or disassortative preferences, in free-ranging males (but not females) mating success varied among colour morphs, with black males mating with more females	Dual and triad choice experiments with different setups in the laboratory, combined with estimates of mating success in free-ranging individuals based on mark recapture study and dusting of individuals with fluorescent coloured powder	Caesar *et al.* [5]
Mating behaviour	+	NE	NA	Mating duration differed according to wing morph in males but not in females	Wild-caught individuals from one population were brought to the laboratory, assessed for mating duration and kept for egg-laying (*n*=11 females)	Steenman *et al.* [6]
Physiology						
Thermal capacity (heating rates)	NE	+	NA	Darker morphs warmed up faster and attained higher equilibrium temperatures than paler morphs	Laboratory study of body temperatures attained under augmented irradiation	Forsman [7]
Temperature preference	+	+	NA	Selected body temperatures were higher in females than males, and varied among colour morphs in females (darker morphs selected higher temperatures that correlated with heating capacity) but not in males	Observations of positioning of wild caught individuals in a laboratory thermal gradient	Forsman [8]
Temperature preference	NA	-/+	NA	No difference in behaviour (positioning in thermal arena) among natural colour morphs, but white-painted individuals used warmer parts of the arena compared with black-painted individuals	Laboratory experiment in which the behaviour (microhabitat choice) of individuals in a thermal (illuminated) mosaic arena was recorded and compared between individuals representing different (pale, intermediate or dark) natural colour morphs and between individuals that had been	Wennersten *et al.* [9]

					painted black or white	
Jumping performance	-	NE	NA	Jumping performance increased with temperature but was independent of sex, despite that females were larger than males	Laboratory experiment and performance trials in climate chamber	Forsman [10]
Jumping performance and reaction distance	NA	+	NA	Jumping capacity increased and reaction distance decreased with body temperature and also varied according to colour morph	Performance trials at two temperatures in climate chamber	Forsman [11]
Whole body fat content	+	-	NA	Relative fat content was higher in females than in males and varied according to time of season, but did not differ in experimental individuals between pale and dark colour morphs or according to sun-exposure	Relative fat content was quantified and compared between wild caught individuals, and between experimental captive individuals maintained in either sunny or shaded conditions	Forsman [12]
Energy reserves and energy consumption	+	+	NA	Energy consumption and protein content was higher in the macropterous than in the brachypterous morph and higher in males than in females, but there were no differences in carbohydrate or lipid contents	Energy reserves (carbohydrates, proteins and lipids) and energy consumption (based on electron transport activity) were quantified and compared between macropterous and brachypterous males and females	Lock *et al.* [13]
Morphology						
Body size	+	+	NA	Females were larger than males, body size varied among colour morphs	Mark-recapture study	Forsman and Appelqvist [1]
Body size	+	+	-	Body size differed between sexes and colour morphs but not between populations	Data for wild-caught individuals from two populations	Forsman [14]
Body size	+	NE	+	Body size differed between populations and between males and females within populations	Wild-caught males and females from two natural populations were compared for body size	Tinnert *et al.* [15]
Body size	+	NE	+	Body size (based on femur length) varied among populations, and between sexes within populations, in both species, but the differences seen in *T. subulata* did not parallel those seen in	Data on body size, the incidence of macropterous flight capable phenotypes, and neutral genetic diversity based	Tinnert and Forsman [16]

				T. undulata	on AFLP (amplified fragment length polymorphism) were compared between *T. subulata* and *T. undulata* from seven sampling locations where the two species were sympatric	
Body size	+	NA	NA	Females were larger than males, and short-winged brachypterous individuals were smaller (had shorter femurs) than long-winged individuals	Body size of wild-caught individuals from one population was compared	Steenman *et al.* [17]
Body size	+	NE	NE	Females were larger than males but there was no difference in body size (femur length) between long- and short-winged individuals	Comparisons of body size based on data for wild-caught individuals from one population	Berggren *et al.* [18]
Body size	+	NE	NE	Females were larger than males but there was no difference in body size (femur length) between long- and short-winged individuals	Comparisons of body size based on re-analyses of data from Tinnert *et al.* [15] for wild-caught individuals from one population	*This study*
Frequency of long-winged flight capable phenotypes	-	NE	+	The incidence of the macropterous long-winged morph is correlated in samples of males and females from different populations, is higher and changes faster between years in disturbed than in stable habitats, is genetically determined and not influenced by plasticity	Comparisons based on data for wild-caught individuals from 13 populations combined with common garden rearing experiments, mother-offspring resemblance analyses	Berggren *et al.* [18]
Frequency of long-winged flight capable phenotypes	-	NE	+	The incidence of the long-winged macropterous morph varied among populations but did not differ significantly between males and females	Wild-caught individuals from ten populations were classified for sex and wing-morph	Steenman *et al.* [17]
Frequency of long-winged flight capable phenotypes	NE	-	NA	The incidence of the long-winged macropterous morph was similar in melanic (black) and non-melanic individuals	Wild-caught individuals from one recently established population in a post fire environment were classified for colour morph and wing-morph	Forsman *et al.* [19]
Frequency of long-winged flight capable phenotypes	-	-	+	The incidence of the long-winged macropterous morph in captive reared families was independent of maternal colour morph but varied among populations and according to maternal wing morph	Re-analyses of data for captive reared *T. subulata* families from 10 populations from Berggren *et al.* [18]	*This study, Figure S3*
Relative frequency of	+	NA	NA	Distribution of individuals among colour morphs	Mark-recapture study	Forsman and Appelqvist [1]

different colour morphs				depend on sex		
Relative frequency of different colour morphs	+	NA	NA	Relative frequencies of different colour morphs changed between years and varied between males and females. Colour morph diversity also changed between years and varied among families according to maternal colour morph	Two years of data for wild-caught and captive reared individuals from one population	Karlsson *et al.* [20]
Relative frequency of different colour morphs	+	NA	+	The relative frequencies of black, striped and grey colour morphs varied between natural populations and were different in males and females	Estimates of colour morph frequencies in males and females based on data for wild-caught individuals from two natural populations	Karpestam *et al.* [21]
Relative frequency of different colour morphs in captive reared individuals	NE	-	NA	The colour morphs and overall darkness of captive reared individuals resembled their mothers but was not influenced by rearing substrate	Offspring born to wild-caught females were reared in solitude using a split-brood design on either crushed charcoal or white aquaria gravel	Karlsson *et al.* [22]
Relative frequency of different colour morphs in captive reared individuals	NE	-	NA	Colour patterns of captive reared individuals were independent of rearing density but strongly influenced by maternal colour pattern	Offspring were experimentally reared either in solitude, at intermediate density or under crowded conditions	Karlsson and Forsman [23]
Relative frequency of different colour morphs in captive reared individuals	NE	+	+	The incidence of the black melanic colour morph was correlated in males and females across samples from different populations, higher in populations in recently burned than in non-burned areas, decreased over time in post-fire environments, and was correlated among samples of captive reared and wild-caught parental generation individuals from corresponding populations	Comparisons of colour morph frequencies, with particular emphasis on the incidence of black/melanic phenotypes, within and between 20 natural populations in unaffected and post-fire environments, combined with common garden captive rearing of six populations	Forsman *et al.* [19]
Relative frequency of different colour morphs in captive reared individuals	NE	+	NA	Results (based on data for only 14 surviving individuals) indicated that whereas colour pattern itself was not influenced by plasticity, individuals exposed to dark backgrounds developed darker colours	Hatchlings from wild-caught individuals from one population were reared in captivity, in groups of non-marked individuals, on either dark or light substrates using a split-brood design	Hochkirch *et al.* [24]

Offspring colour morph diversity	NA	+	NA	Colour morph diversity of offspring did not differ between families produced by monandrous and polyandrous females, mother-offspring colour morph resemblance was higher when the father(s) belonged to the same morph as the mother	Females were experimentally mated with one of four males of the same or different colour morphs, followed by captive rearing of resulting eggs and offspring	Caesar and Forsman [25]
Offspring colour morph diversity	NA	+	NA	Females mated with multiple males produce half-sibling offspring (sired by different males) that were more colour morph diverse	Offspring produced by un-manipulated wild-caught females and by captive reared virgin females from 12 populations that had been experimentally mated with different numbers of males, were analysed for paternity using microsatellite markers and assessed for colour morph diversity	Johansson *et al.* [26]
Life-history						
Clutch size and egg size	NA	-	-	Clutch size and egg size did not vary according to colour morph or population	Data for wild-caught individuals from two populations kept in laboratory for egg laying	Forsman [14]
Trade-off between clutch size and egg size	NA	+	NE	The relationship between clutch size and egg size varied according to colour morph	Data for wild-caught individuals from two populations kept in laboratory for egg laying	Forsman [14]
Inter-clutch interval	NA	+	NE	Time interval between sequential clutches varied among colour morphs	Data for wild-caught individuals from two populations kept in laboratory for egg laying	Forsman [14]
Clutch size	NA	-	NA	Clutch size did not differ between colour morphs or between temperature treatments, but females in warm treatment produced more clutches	Wild-caught females were maintained in laboratory at two temperatures for egg laying	Forsman [12]
Clutch size and hatching success of eggs	NA	NE	+	Clutch size and hatching success of eggs produced by wild-caught females differed according to source population	Wild caught males and females from two natural populations were experimentally either purebred	Tinnert *et al.* [15]

				or interbred (admixed) in captivity and clutches ere incubated in the laboratory	
Inter-clutch interval	NA	+	NA	Time interval between sequential clutches varied among colour morphs	Wild caught females maintained in laboratory at two temperatures for egg laying	Forsman [12]
Clutch size	NA	NE	+	Clutch size varied among females originating from different populations, but there was no consistent difference in clutch size or inter-clutch interval between macropterous (long-winged) and macropterous (short-winged) females	Comparisons based on data for wild-caught individuals from 13 populations combined with common garden rearing experiments, mother-offspring resemblance analyses, and behavioural assays	Berggren *et al.* [18]
Clutch size (number of hatched nymphs)	NA	NE	+	Number of hatched nymphs per clutch varied depending on maternal source population	Wild-caught females from five natural populations were kept in the laboratory for egg-laying	Wennersten *et al.* [27]
Mating behaviour and egg size	+	NE	NA	Mating duration differed according to wing morph in males but not in females, short-winged females produced similar clutch sizes but smaller eggs than long-winged females	Wild-caught individuals from one population were brought to the laboratory, assessed for mating duration and kept for egg-laying (*n*=11 females)	Steenman *et al.* [6]
Performance						
Survival of offspring	NA	+	NA	Parental colour morph resemblance increased viability of offspring (indicative of compatibility effects), and monandrous females produced offspring that survived better compared with offspring produced by polyandrous females (under shaded bot not under sunny conditions)	Females were experimentally mated with one of four males of the same or different colour morphs, followed by incubation of resulting eggs and captive rearing of offspring in sun-exposed or shaded conditions	Caesar and Forsman [25]
Survival of offspring	NA	+	NA	Survival was enhanced by relatedness, and survival increased with increasing colour morph diversity (under high but not under low density)	Newly hatched nymphs in experimental groups originating from one, three or seven different mothers were	Caesar *et al.* [28]

					reared in captivity under high and low density	
Survival	+	+	NA	Differences in survival among manipulated colour morph were different in males and females	Mark-recapture study combined with manipulation of colour patterns in the wild	Forsman and Appelqvist [1]
Susceptibility to visual predators (birds)	NE	+	NE	Direction of difference between colour morphs in survival and escape performance changed with temperature	Predation experiment using domesticated chickens at two temperatures in climate chamber	Forsman and Appelqvist [29]
Susceptibility to visual predators (lizards)	NE	-	NA	No difference in survival of dark and pale individuals when exposed to predation by lizards	Predation experiment using lizards in shaded and sun-exposed cages	Civantos *et al.* [30]
Detection rate / predation risk	NA	+	NA	Detectability, the protective value of black coloration, gradually increases in habitats that have been more blackened by fire	Estimation of detection rate of grasshopper images representing the melanic black colour morph presented in sequence to human ‘predators’ against photographic samples of natural visual backgrounds on computer screens	Karpestam *et al.* [31]
Detection rate / predation risk	NA	+	NA	The proportion of grasshoppers that were detected, and time to detection, depended on colour pattern and the type of visual background	Estimation of detection rate of grasshopper images representing different colour morphs (black, striped and grey) presented to human ‘predators’ against photographic samples of natural visual backgrounds (unburned, intermediate, and heavily burned) on computer screens	Karpestam *et al.* [32]
Detection rate / predation risk	NA	+	NA	Fewer grasshoppers were detected when presented in mixed than in uniform sequences, and all three morphs benefitted from being presented in mixed sequences	Estimation of detection rate of grasshopper images representing different colour morphs (black, striped and grey) presented to human ‘predators’ against photographic samples of natural visual backgrounds	Karpestam *et al.* [33]

					(representing semi-burnt habitats) on computer screens in either polymorphic or monomorphic sequences	
Detection rate / predation risk	+	+	NA	Colour pattern and body size interactively influenced rates of detection, and the relative frequencies of colour morphs in natural populations differed between sexes	Detection rates were compared between large (representing females), intermediate, and small (representing males) images of black, striped, and grey grasshoppers presented against natural visual backgrounds on computer screens to human 'predators'. Results compared with estimates of morph frequencies in males and females in two natural populations	Karpestam *et al.* [21]
Detection rate / predation risk	NA	+	NA	Polymorphism afforded protection against predators, for both individuals and populations, by reducing the rates of detection compared with monomorphism, and the benefits of polymorphism decreased with increasing relative camouflage	Estimation of detection rate of grasshopper images (black, striped, grey, brown or barred morphs) presented in groups that represented four levels of colour pattern polymorphism to human 'predators' against photographic samples of natural visual backgrounds (representing semi-burnt habitats) on computer screens	Karpestam *et al.* [34]
Detection rate / predation risk	+	+	NA	Size-variability either increased or reduced detection depending on prey colour pattern, and the direction of the effect of size variability changed across colour patterns as the bias in perceived size increased	Grasshopper images of different colour pattern (black, striped or grey) were presented on computer screens to human 'predators' in size-variable (small, medium and large) or size-invariable (all medium) sequences against photographs of natural visual backgrounds, and biases in	Karpestam *et al.* [35]

					perceived prey size were assessed using presentation cards showing pairs of grasshoppers	
Establishment success in outdoor enclosures	NA	+	-	More colour morph diverse experimental founder populations produced larger next-generation populations, but there were no effects of source population	Experimental outdoor enclosures were seeded with founder groups that varied with regards to colour morph diversity, and population sizes were assessed one year after introductions	Wennersten *et al.* [27]
Genetics						
Genetic structure and diversity within populations	NE	NE	+	Genetic divergence among populations, and low to moderate diversity within populations, genetic diversity within population increased with population size, no strong signature of isolation by distance	Wild-caught individuals from 20 sampling locations were used for population genetics analyses based on AFLP (amplified fragment length polymorphism) data	Tinnert *et al.* [36]
Genetic differentiation between populations and diversity within populations	NA	NE	+	Genetic divergence among populations and low to moderate diversity within populations in both species, genetic differentiation between pairs of populations was generally lower in the more dispersive *T. subulata* than in *T. undulata*	Data on body size, the incidence of macropterous flight capable phenotypes, and neutral genetic diversity based on AFLP (amplified fragment length polymorphism) were compared between *T. subulata* and *T. undulata* from seven sampling locations where the two species were sympatric	Tinnert and Forsman [16]
TETRIX UNDULATA						
Behaviours						
Microhabitat use	+	+	NA	Grasshoppers differently preferred and utilized different microhabitats (types of substrates) and surface temperatures depending on sex and colour morph. Degree of habitat selectivity varied among colour morphs, being highest in the black and lowest in the striped morph	Behavioural observations of free-ranging individuals in the wild combined with laboratory experiments	Ahnesjö and Forsman [37]

Temperature preferences in the wild	+	+	NA	Grasshoppers selected microhabitats of different thermal quality depending on sex (females selected warmer habitats than males) and colour morph, and avoidance of habitats characterized by high surface temperatures was stronger in dark compared to pale colour morphs	Behavioural observations of free-ranging individuals in the wild	Ahnesjö and Forsman [37]
Temperature preferences in the laboratory	+	+	NA	Selected body temperatures were higher in females than males, higher in individuals reared under low than high temperatures, and varied according to maternal colour morph in females (offspring to darker mothers selected higher temperatures, that correlated with heating capacity) but not in males	Observations of positioning of captive reared individuals in a laboratory thermal gradient	Forsman *et al.* [38]
Temperature regulatory (basking) behaviour	+	+	NA	Males and females, and dark and pale females (but not dark and pale males) differed in basking behaviour (frequency and duration)	Behavioural observations of captive reared individuals in experimental arenas imposing a trade-off between temperature regulation and feeding	Forsman *et al.* [38]
Escape behaviour	NE	+	NA	Colour morphs differently modified their substrate use when subjected to elevated predation risk	Behavioural observations of captive individuals in experimental arenas	Ahnesjö and Forsman [37]
Physiology						
Thermal capacity (heating rates)	NE	+	NA	Darker morphs (natural as well as painted) warmed up faster and attained higher equilibrium temperatures	Laboratory study of body temperatures attained under augmented irradiation	Forsman *et al.* [38]
Developmental instability	NE	+	NA	Developmental instability varied according to colour morph, being higher in darker than in paler morphs	Developmental instability was quantified and compared based on estimates of fluctuating asymmetry of femur size in wild-caught individuals	Civantos *et al.* [39]
Immune defence	-	-	NA	Immune defences was independent of sex and colour morph	Comparisons of encapsulation response to a novel antigen (nylon monofilament)	Civantos *et al.* [40]

Morphology						
Body size	+	+	NA	Body size was larger in females than males, independent of rearing temperature, decreased with increasing time to maturity, varied among colour morphs, and according to maternal colour morph (in warm but not in cold temperature)	Split-brood design captive rearing experiment in either warm or cold temperature	Ahnesjö and Forsman [41]
Body size	+	NE	+	Body size (femur length) varied among populations, and between sexes within populations, in both species, but the differences seen in *T. subulata* did not parallel those seen in *T. undulata*	Data on body size compared between wild-caught *T. subulata* and *T. undulata* from seven sampling locations where the two species were sympatric	Tinnert and Forsman [16]
Relative frequency of different colour morphs in natural populations	NE	+	+	Colour morph diversity was higher in disturbed than in stable environments, and was negatively associated with neutral genetic (AFLP) diversity across populations	Data on colour morph diversity and genetic diversity based on AFLP (amplified fragment length polymorphism) collected for individuals from 20 natural populations in either disturbed or stable environments	Yildirim *et al.* [42], Tinnert [43]
Relative frequency of different colour morphs in captive reared individuals	NE	+	NA	The phenotypic expression of a melanic (black) colour pattern was independent of rearing temperature but was higher in offspring born to darker mothers	Offspring to wild-caught individuals were experimentally reared under cold and warm conditions using a split-brood design	Forsman [44]
Life-history						
No. of hatchlings	NA	-	NA	Number of hatchlings was independent of maternal colour morph	Captive reared females maintained in laboratory for egg laying	Ahnesjö and Forsman [41]
Time to maturity	NE	+	NA	Time to maturity of captive reared individuals was longer in cold than in warm temperature and varied according to maternal colour morph	Split-brood design captive rearing experiment in either warm or cold temperature	Ahnesjö and Forsman [41]
Performances						
Parasite prevalence	-	-	NA	Prevalence of the endoparasitic fly was	Comparison of endoparasitic	Civantos *et al.* [40]

				independent of grasshopper sex and colour morph	fly *Leiophora innoxia* prevalence in wild-caught grasshoppers	
Survival of individuals reared in in captivity under cold and warm temperatures	NE	+	NA	Relative survival of melanic (black) and non-melanic individuals was not affected by rearing temperature but depended on maternal colour morph; with melanic individuals produced by black mothers surviving longer than melanic individuals produced by non-black mothers, and non-melanic individuals produced by non-black mothers surviving longer than those produced by black mothers	Offspring to wild-caught individuals were experimentally reared under cold and warm conditions using a split-brood design	Forsman [44]
Genetic structure and diversity						
Genetic differentiation between populations and diversity within populations	NE	NE	+	Genetic divergence among populations and low to moderate diversity within populations in both species, genetic differentiation between pairs of populations was generally higher in the less dispersive *T. undulata* than in *T. subulata*	Data on genetic diversity based on AFLP (amplified fragment length polymorphism) were compared between *T. subulata* and *T. undulata* from seven sampling locations where the two species were sympatric	Tinnert and Forsman [16]
Neutral and functional genetic diversity within populations	+	NE	+	Within population neutral genetic diversity was generally greater in stable than in disturbed environments, whereas functional (outlier) diversity was slightly lower in stable environments, neutral diversity increased with increasing proportion long-winged phenotypes across populations, and functional (outlier AFLP) genetic structure differed between males and females	Data on colour morph diversity, the incidence of macropterous flight capable phenotypes, and neutral and functional (outlier loci) genetic diversity based on AFLP (amplified fragment length polymorphism) collected for 20 natural populations from either disturbed or stable environments	Yildirim *et al.* [42], Tinnert [43], *This study* (see Table S2)

+ denotes that a statistically significant difference was detected between groups (sexes, colour morphs or populations)

-- denotes no significant difference detected, NA denotes not applicable, NE denotes not evaluated

Supporting References

1. Forsman A., Appelqvist S. 1999. Experimental manipulation reveals differential effects of colour pattern on survival in male and female pygmy grasshoppers. *J Evol Biol* **12**, 391-401.
2. Karpestam E., Wennersten L., Forsman A. 2012. Matching habitat choice by experimentally mismatched phenotypes. *Evol Ecol* **26**, 893-907.
3. Karpestam E., Forsman A. 2011. Dietary differences among colour morphs of pygmy grasshoppers revealed by behavioural experiments and stable isotopes. *Evol Ecol Res* **13**, 461-477.
4. Karpestam E., Forsman A. 2013. Stable isotopes reveal dietary divergence between dispersal phenotypes in *Tetrix subulata* pygmy grasshoppers. *Euro J Entomol* **110**, 65-70.
5. Caesar S., Ahnesjö J., Forsman A. 2007. Testing the role of co-adapted genes versus bet hedging for mating strategies in colour polymorphic pygmy grasshoppers. *Biol J Linn Soc* **90**, 491-499.
6. Steenman A., Lehmann A.W., Lehmann G.U.C. 2015. Life-history trade-off between macroptery and reproduction in the wing-dimorphic pygmy grasshopper *Tetrix subulata* (Orthoptera Tetrigidae). *Ethol Ecol Evol* **27**, 93-100.
7. Forsman A. 1997. Thermal capacity of different colour morphs in the pygmy grasshopper *Tetrix subulata*. *Ann Zool Fenn* **34**, 145-149.
8. Forsman A. 2000. Some like it hot: Intra-population variation in behavioral thermoregulation in color-polymorphic pygmy grasshoppers. *Evol Ecol* **14**, 25-38.
9. Wennersten L., Karpestam E., Forsman A. 2012. Phenotype manipulation influences microhabitat choice in pygmy grasshoppers. *Curr Zool* **58**, 392-400.
10. Forsman A. 1999. Temperature influence on escape behaviour in two species of pygmy grasshoppers. *Ecoscience* **6**, 35-40.
11. Forsman A. 1999. Variation in thermal sensitivity of performance among colour morphs of a pygmy grasshopper. *J Evol Biol* **12**, 869-878.
12. Forsman A. 2001. Clutch size versus clutch interval: life history strategies in the colour-polymorphic pygmy grasshopper *Tetrix subulata*. *Oecologia* **129**, 357-366.
13. Lock K., Verslycke T., Janssen C.R. 2006. Energy allocation in brachypterous versus macropterous morphs of the pygmy grasshopper *Tetrix subulata* (Orthoptera : Tetrigidae). *Entomol Gener* **28**, 269-274.
14. Forsman A. 1999. Reproductive life history variation among colour morphs of the pygmy grasshopper *Tetrix subulata*. *Biol J Linn Soc* **67**, 247-261.
15. Tinnert J., Berggren H., Forsman A. 2016. Population-specific effects of interbreeding and admixture on reproductive decisions and offspring quality. *Ann Zool Fennici* **53**, 55-68.
16. Tinnert J., Forsman A. 2017. The role of dispersal for genetic and pehnotypic variation: insights from comparisons of sympatric pygmy grasshoppers. *Biol J Linn Soc* **blx055**, 84-97.

17. Steenman A., Lehmann A.W., Lehmann G.U.C. 2013. Morphological variation and sex-biased frequency of wing dimorphism in the pygmy grasshopper *Tetrix subulata* (Orthoptera: Tetrigidae). *Euro J Entomol* **110**, 535-540.
18. Berggren H., Tinnert J., Forsman A. 2012. Spatial sorting may explain evolutionary dynamics of wing polymorphism in pygmy grasshoppers. *J Evol Biol* **25**, 2126-2138. doi: 2110/1111/j.1420-9101.2012.02592.x.
19. Forsman A., Karlsson M., Wennersten L., Johansson J., Karpestam E. 2011. Rapid evolution of fire melanism in replicated populations of pygmy grasshoppers. *Evolution* **65**, 2530-2540.
20. Karlsson M., Caesar S., Ahnesjö J., Forsman A. 2008. Dynamics of colour polymorphism in changing environments: Fire melanism and then what? *Oecologia* **154**, 715-724.
21. Karpestam E., Merilaita S., Forsman A. 2014. Body size influences differently the detectabilities of colour morphs of cryptic prey. *Biol J Linn Soc* **113**, 112-122.
22. Karlsson M., Johansson J., Caesar S., Forsman A. 2009. No evidence for developmental plasticity of color patterns in response to rearing substrate in pygmy grasshoppers. *Can J Zool* **87**, 1044-1051.
23. Karlsson M., Forsman A. 2010. Is melanism in pygmy grasshoppers induced by crowding? *Evol Ecol* **24**, 975-983.
24. Hochkirch A., Depperman J., Gröning J. 2008. Phenotypic plasticity in insects: The effects of substrate colour on the colouration of two ground-hopper species. *Evol Dev* **10**, 350-359.
25. Caesar S., Forsman A. 2009. Do polyandrous pygmy grasshopper females obtain fitness benefits for their offspring? *Behav Ecol* **20**, 354-361.
26. Johansson J., Caesar S., Forsman A. 2013. Multiple paternity increases phenotypic diversity in *Tetrix subulata* pygmy grasshoppers. *J Orthopt Res* **22**, 79-85.
27. Wennersten L., Johansson J., Karpestam E., Forsman A. 2012. Higher establishment success in more diverse groups of pygmy grasshoppers under seminatural conditions. *Ecology* **93**, 2519-2525.
28. Caesar S., Karlsson M., Forsman A. 2010. Diversity and relatedness enhance survival in colour polymorphic grasshoppers. *PLoS ONE* **5**, e10880.
29. Forsman A., Appelqvist S. 1998. Visual predators impose correlational selection on prey color pattern and behavior. *Behav Ecol* **9**, 409-413.
30. Civantos E., Ahnesjö J., Forsman A., Martin J., Lopez P. 2004. Indirect effects of prey coloration on predation risk: pygmy grasshoppers versus lizards. *Evol Ecol Res* **6**, 201-213.
31. Karpestam E., Merilaita S., Forsman A. 2012. Reduced predation risk for melanistic pygmy grasshoppers in post-fire environments. *Ecol & Evol* **2**, 2204-2212.
32. Karpestam E., Merilaita S., Forsman A. 2013. Detection experiments with humans implicate visual predation as a driver of colour polymorphism dynamics in pygmy grasshoppers. *BMC Ecology* **13:17**.

33. Karpestam E., Merilaita S., Forsman A. 2014. Natural levels of colour polymorphism reduce performance of visual predators searching for camouflaged prey. *Biol J Linn Soc* **112**, 546-555.
34. Karpestam E., Merilaita S., Forsman A. 2016. Colour polymorphism protects prey individuals and populations against predation. *Scientific Reports* **6**, 22122.
35. Karpestam E., Merilaita S., Forsman A. 2018. Size variability effects on visual detection are influenced by colour pattern and perceived size. *Anim Behav* ***in press*.**
36. Tinnert J., Hellgren O., Lindberg J., Koch-Schmidt P., Forsman A. 2016. Population genetic structure, differentiation and diversity in *Tetrix subulata* pygmy grasshoppers: roles of population size and immigration. *Ecol & Evol* **6**, 7831-7846.
37. Ahnesjö J., Forsman A. 2006. Differential habitat selection by pygmy grasshopper color morphs; interactive effects of temperature and predator avoidance. *Evol Ecol* **20**, 235-257.
38. Forsman A., Ringblom K., Civantos E., Ahnesjö J. 2002. Coevolution of color pattern and thermoregulatory behavior in polymorphic pygmy grasshoppers *Tetrix undulata*. *Evolution* **56**, 349-360.
39. Civantos E., Forsman A., Ahnesjö J. 2005. Developmental instability and immune function in colour polymorphic pygmy grasshoppers. *Evol Ecol* **19**, 1-14.
40. Civantos E., Ahnesjö J., Forsman A. 2005. Immune function, parasitization and extended phenotypes in colour polymorphic pygmy grasshoppers. *Biol J Linn Soc* **85**, 373-383.
41. Ahnesjö J., Forsman A. 2003. Correlated evolution of colour pattern and body size in polymorphic pygmy grasshoppers, *Tetrix undulata*. *J Evol Biol* **16**, 1308-1318.
42. Yildirim Y., Tinnert J., Forsman A. 2018. Contrasting patterns of neutral and funtional genetic diversity in stable and disturbed environments. *Unpublished manuscript*.
43. Tinnert J. 2017. *Microevolution in pygmy grasshoppers*. Växjö, Linnaeus University Press; 45 p.
44. Forsman A. 2011. Rethinking the thermal melanism hypothesis: rearing temperature and coloration in pygmy grasshoppers. *Evol Ecol* **25**, 1247-1257.
