# Supplementary material for: On the role of sex differences for evolution in heterogeneous and changing fitness landscapes: insights from pygmy grasshoppers
Source: Philos Trans R Soc Lond B Biol Sci. 2018 Aug 27;373(1757):20170429. doi: 10.1098/rstb.2017.0429 (PMC6125723; doi:10.1098/rstb.2017.0429)
Supplement: Table S2 [file rstb20170429supp2.pdf]

Table S2. Genetic structure of outlier genetic loci in *Tetrix undulata* according to sampling location and sex. Analysis of amplified fragment length polymorphism (AFLP) [1, 2] was carried out using data for 323 individuals representing 20 sampling locations. A Bayesian approach implemented in BayeScan V.2.1 software [3] detected 28 AFLP outlier loci influenced by selection. Data on the outlier AFLP loci were analysed using permutational multivariate analysis of variance (PERMANOVA) [4, 5] to test for differences in genetic structures among different locations (treated as a random factor) and between sexes (fixed factor). Permutation tests used 9,999 unrestricted permutations and partial sums of squares type (Type III).

Source of variation	*df*	SS	MS	Pseudo-*F*	*P*(perm)	Unique permutations	*P*(MC)
Population	19	14414	758.62	17.244	0.0001	9846	0.0001
Sex	1	179.93	179.93	4.09	0.0057	9956	0.0082
Residual	303	13330	43.993				
Total	323	28206					

Supporting References

1. Bensch S., Åkesson M. 2005. Ten years of AFLP in ecology and evolution: why so few animals. *Mol Ecol* **14**, 2899-2914.
2. Vos P., Hogers R., Bleeker M., Reijans M., van de Lee T., Hornes M., Frijters A., Pot J., Peleman J., Kuiper M., et al. 1995. AFLP: a new technique for DNA fingerprinting. *Nucleic Acids Research* **23**, 4407-4414.
3. Foll M., Gaggiotti O. 2008. A genome-scan method to identify selected loci appropriate for both dominant and codominant markers: A Bayesian perspective. *Genetics* **180**, 977-993.
4. Anderson M.J. 2001. A new method for non-parametric multivariate analysis of variance. *Austral Ecol* **26**, 32-46.
5. McArdle B.H., Anderson M.J. 2001. Fitting multivariate models to community data: a comment on distance based redundancy analysis. *Ecology* **82**, 290-297.
