# Supplementary material for: On the role of sex differences for evolution in heterogeneous and changing fitness landscapes: insights from pygmy grasshoppers
Source: Philos Trans R Soc Lond B Biol Sci. 2018 Aug 27;373(1757):20170429. doi: 10.1098/rstb.2017.0429 (PMC6125723; doi:10.1098/rstb.2017.0429)
Supplement: Figure S1 [file rstb20170429supp3.pdf]

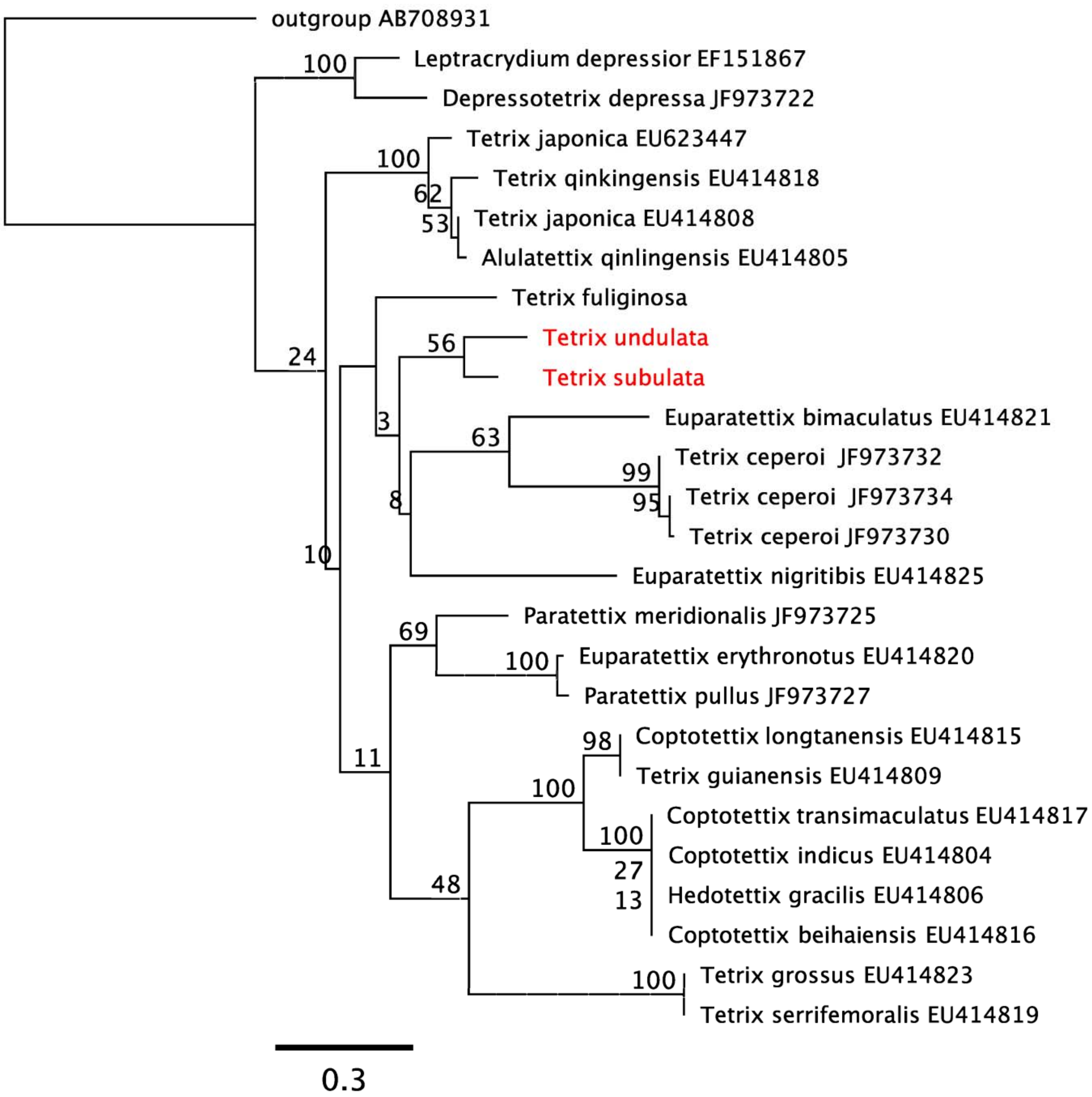

Figure S1. Maximum-Likelihood tree (GTR+I model of evolutionary changes) of species selected to provide a broad phylogenetic and geographic coverage of pygmy grasshoppers. Numbers of the branches represent bootstrap values based on 100 iterations. The phylogeny is based on 285bp of the cytochrome oxidase I (COI) mtDNA gene. Reference sequences where obtained from GenBank with the accession numbers shown after the name in the phylogeny. *Tetrix undulata* and *T. subulata* represent sister taxa, but although grouping together in the phylogeny the genetic distance is rather high (p-distance: 7.4% based on the full 396bp). Analyses were performed by Olof Hellgren.
