# Supplementary material for: On the role of sex differences for evolution in heterogeneous and changing fitness landscapes: insights from pygmy grasshoppers
Source: Philos Trans R Soc Lond B Biol Sci. 2018 Aug 27;373(1757):20170429. doi: 10.1098/rstb.2017.0429 (PMC6125723; doi:10.1098/rstb.2017.0429)
Supplement: Figure S2 [file rstb20170429supp4.pdf]

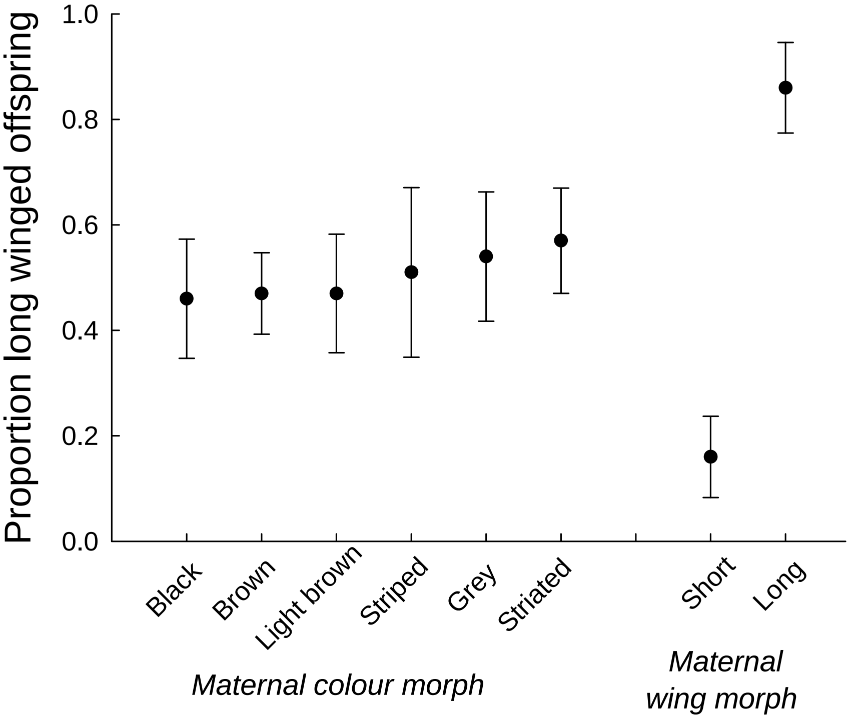

Figure S2. Proportion long-winged *Tetrix subulata* phenotypes in families reared in captivity under controlled conditions is independent of maternal colour morph but differ depending on maternal wing morph. Results are based on analyses of data (from Berggren *et al.* [1]) for 1785 adult captive reared offspring, representing 212 families from 10 natural populations. Data analysed using generalized linear mixed models implemented using procedure GLIMMIX in SAS, individuals rather than family means were used as data points, maternal colour morph and maternal wing morph were treated as fixed effects, source population was treated as a random effect: effect of colour morph: $F_{5,212} = 0.70$, $P = 0.62$; effect of wing morph: $F_{1,212} = 205.76$, $P < 0.0001$; covariance parameter estimate associated with random effect of population was highly significant: 3.90 ± 0.60). Figure shows least-squares means ± 95%CI.
